# Supplementary material for: Development of chemokine network inhibitors using combinatorial saturation mutagenesis
Source: Commun Biol. 2025 Apr 3;8:549. doi: 10.1038/s42003-025-07778-6 (PMC11969024; doi:10.1038/s42003-025-07778-6)
Supplement: Supplementary file 4 — Supplementary Data 2 [file 42003_2025_7778_MOESM4_ESM.zip › SupplementaryData3/scripts/xNNKi.tar!/./script/analysis/JK/ExactPvalues.docx]

Supplementary Table X: Exact P values for phage-display mutagenesis analyses,
comparisons to the control

| Residue | Selection method | Number of samples | P value |
| --- | --- | --- | --- |
| HD2 | selected ALL | 22 | NA |
| E1D | selected ALL | 22 | 1.06e-01 |
| E2D | selected ALL | 22 | 7.66e-01 |
| E2W | selected ALL | 22 | 1.00e+00 |
| Y5W | selected ALL | 22 | 9.14e-02 |
| T6D | selected ALL | 22 | 1.57e-04 |
| T6W | selected ALL | 22 | 1.81e-04 |
| A7D | selected ALL | 22 | 4.77e-02 |
| Y8W | selected ALL | 22 | 9.99e-01 |
| A9W | selected ALL | 22 | 3.78e-01 |
| P10D | selected ALL | 22 | 1.09e-05 |
| L11I | selected ALL | 22 | 9.26e-03 |
| T12I | selected ALL | 22 | 7.54e-01 |
| T12V | selected ALL | 22 | 7.62e-01 |
| T12W | selected ALL | 22 | 3.15e-01 |
| T16C | selected ALL | 22 | 4.76e-02 |
| T16D | selected ALL | 22 | 5.51e-01 |
| HD2 | selected CC | 15 | NA |
| E1D | selected CC | 15 | 9.90e-02 |
| E2D | selected CC | 15 | 8.17e-01 |
| E2W | selected CC | 15 | 6.72e-01 |
| Y5W | selected CC | 15 | 6.29e-01 |
| T6D | selected CC | 15 | 1.40e-03 |
| T6W | selected CC | 15 | 7.76e-02 |
| A7D | selected CC | 15 | 3.65e-01 |
| Y8W | selected CC | 15 | 3.80e-01 |
| A9W | selected CC | 15 | 1.48e-04 |
| P10D | selected CC | 15 | 1.08e-12 |
| L11I | selected CC | 15 | 3.19e-02 |
| T12I | selected CC | 15 | 9.88e-01 |
| T12V | selected CC | 15 | 1.00e+00 |
| T12W | selected CC | 15 | 1.89e-01 |
| T16C | selected CC | 15 | 5.17e-06 |
| T16D | selected CC | 15 | 9.94e-01 |
| HD2 | selected CXnC | 7 | NA |
| E1D | selected CXnC | 7 | 7.50e-01 |
| E2D | selected CXnC | 7 | 9.85e-01 |
| E2W | selected CXnC | 7 | 9.16e-01 |
| Y5W | selected CXnC | 7 | 4.19e-02 |
| T6D | selected CXnC | 7 | 1.36e-02 |
| T6W | selected CXnC | 7 | 2.76e-05 |
| A7D | selected CXnC | 7 | 4.37e-02 |
| Y8W | selected CXnC | 7 | 8.84e-01 |
| A9W | selected CXnC | 7 | 1.55e-01 |
| P10D | selected CXnC | 7 | 9.97e-01 |
| L11I | selected CXnC | 7 | 1.15e-01 |
| T12I | selected CXnC | 7 | 6.51e-01 |
| T12V | selected CXnC | 7 | 3.99e-01 |
| T12W | selected CXnC | 7 | 9.94e-01 |
| T16C | selected CXnC | 7 | 4.66e-01 |
| T16D | selected CXnC | 7 | 2.42e-01 |
